# Response of marine bacteria to oil contamination and to high pressure and low temperature deep sea conditions

**DOI:** 10.1002/mbo3.550

**Published:** 2017-10-23

**Authors:** Hanna Fasca, Livia V. A. de Castilho, João Fabrício M. de Castilho, Ilson P. Pasqualino, Vanessa M. Alvarez, Diogo de Azevedo Jurelevicius, Lucy Seldin

**Affiliations:** ^1^ Laboratório de Genética Microbiana Instituto de Microbiologia Paulo de Góes Universidade Federal do Rio de Janeiro Rio de Janeiro Brazil; ^2^ Laboratório de Tecnologia Submarina/PENO/COPPE Universidade Federal do Rio de Janeiro Rio de Janeiro Brazil

**Keywords:** bacterial communities, deep sea, oil contamination, pressure, seawater, temperature

## Abstract

The effect of pressure and temperature on microbial communities of marine environments contaminated with petroleum hydrocarbons is understudied. This study aims to reveal the responses of marine bacterial communities to low temperature, high pressure, and contamination with petroleum hydrocarbons using seawater samples collected near an offshore Brazilian platform. Microcosms containing only seawater and those containing seawater contaminated with 1% crude oil were subjected to three different treatments of temperature and pressure as follows: (1) 22°C/0.1 MPa; (2) 4°C/0.1 MPa; and (3) 4°C/22 MPa. The effect of depressurization followed by repressurization on bacterial communities was also evaluated (4°C/22 MPaD). The structure and composition of the bacterial communities in the different microcosms were analyzed by PCR‐DGGE and DNA sequencing, respectively. Contamination with oil influenced the structure of the bacterial communities in microcosms incubated either at 4°C or 22°C and at low pressure. Incubation at low temperature and high pressure greatly influenced the structure of bacterial communities even in the absence of oil contamination. The 4°C/22 MPa and 4°C/22 MPaD treatments resulted in similar DGGE profiles. DNA sequencing (after 40 days of incubation) revealed that the diversity and relative abundance of bacterial genera were related to the presence or absence of oil contamination in the nonpressurized treatments. In contrast, the variation in the relative abundances of bacterial genera in the 4°C/22 MPa‐microcosms either contaminated or not with crude oil was less evident. The highest relative abundance of the phylum Bacteroidetes was observed in the 4°C/22 MPa treatment.

## 1 INTRODUCTION

Seawater comprises more than 70 percent of the Earth's surface. Despite the deep sea being the largest ecosystem on Earth and presenting enormous biodiversity, it is poorly studied (Ramirez‐Llodra et al., 2011). Technological development has increased the exploration and exploitation of deep sea goods and services (Benn et al., 2010), as exemplified by progress in offshore oil exploration by petroleum industries. However, acquisition of scientific knowledge on the microbiology of marine ecosystems, mainly how the microbial community is shaped in deep sea, has not followed this development (Cordes et al., 2016). Only recently, Frank, Garcia, Herndl, and Reinthaler (2016) demonstrated that the prokaryotic diversity in deep‐water masses in the eastern North Atlantic is determined by the connectivity between surface and deep waters, in the same way that Hubalek et al. (2016) suggested that the connectivity to the surface is one of the major drivers of microbial community composition in a deep terrestrial subsurface aquifer of the Fennoscandian shield.

Oil industries are responsible for marine pollution due to chronic contamination from oil discharges from shipping, as a result of tank cleaning or loading, and oil spills. Surface‐water spills form thin layers that partially dissolve, emulsify, and diffuse through the water column, reaching the deep sea (Tkalich, Huda, & Hoong Gin, 2003) and sinking due to the formation of heavier particles (Parinos et al., 2013). In the largest marine oil spill in history, 779 million liters of oil were released into the Gulf of Mexico when the Deepwater Horizon (DWH) drilling rig platform (located at ∼1500 m below surface level) exploded (Atlas & Razen, 2011). Currently, there are thousands of large‐scale oil and gas platforms around the world (Ronconi, Allard, & Taylor, 2015), which have the risk of causing environmental disasters.

Nutrient input (mainly carbon sources ‐ oil hydrocarbons) in marine environments controls bacterial abundance and changes in bacterial community structure (Scoma et al., 2016; Valentine et al., 2012), including for the hydrocarbon‐degrading microorganisms that mediate biodegradation and naturally remediate oil contamination (Leahy & Colwell, 1990). The most abundant bacteria found in marine oil‐contaminated environments are typically Gammaproteobacteria (Head, Jones, & Röling, 2006). Bacterial dominance shifts substantially over time resulting in the decrease in total bacterial diversity (Kleindienst et al., 2015). For example, initial enrichment in Oceanospirillales and *Pseudomonas* shifted over time to dominance of *Colwellia*,* Cycloclasticus,* and *Pseudoalteromonas* following DWH (Dubinsky et al., 2013; Kleindienst et al., 2015; Rivers et al., 2013).

Other environmental parameters (mainly increased hydrostatic pressure and low temperature) may specifically influence the presence and activity of bacterial communities. Low temperature is an important factor that can reduce bacterial richness and catabolic diversity (Meng, Liu, Bao, & Sun, 2016). Moreover, temperature has a direct effect on microbial physiology and also has an effect on the physical properties of oil, influencing its bioavailability (Coulon, McKew, Osborn, McGenity, & Timmis, 2007; Venosa & Holder, 2007). Two bacterial groups, Oceanospirillales and *Colwellia*, known as psychrophiles, were found in DWH samples. In particular, *Colwellia* was much more abundant in crude oil enrichments at 4°C than at room temperature, suggesting that temperature played a significant role (Redmond & Valentine, 2012).

Only a few studies regarding bacteria in marine oil‐contaminated environments under high‐pressure conditions have been performed (Grossi et al., 2010; Schedler, Hiessl, Valladares Juárez, Gust, & Müller, 2014). Oil biodegradation has been extensively investigated at atmospheric pressure of 0.1 MPa, including studies performed using DWH samples (Atlas & Razen, 2011; Gutierrez et al., 2013; Joye, Teske, & Kostka, 2014; Redmond & Valentine, 2012; Scoma et al., 2016; Tapilatu et al., 2010; Yergeau et al., 2015; and many others), probably because of the lack of specialized high‐pressure lab technology. Using high‐pressure reactors, a progressive decrease in the degradation rate of hexadecane was observed in two strains (*Rhodococcus qingshengii* and *Sphingobium yanoikuyae*) as pressure increased (Schedler et al., 2014). More recently, Scoma et al. (2016) demonstrated that two strains belonging to two different species of the genus *Alcanivorax* (*A. jadensis* KS_339 and *A*. *dieselolei* KS_293), which are known to grow rapidly after oil spills, showed significantly lower growth yields under pressures of 5 and 10 MPa.

Nevertheless, studies related to bacterial communities in extreme deep‐water conditions—that simultaneously consider low temperature, high pressure, and oil contamination—are less frequent. Therefore, this study aims to improve our understanding about the shifts in bacterial community structure and composition in response to oil contamination under the effects of temperature and pressure (using high‐pressure lab technology) representing deep sea conditions, using molecular approaches such as PCR‐DGGE and high‐throughput sequencing.

## 2 MATERIALS AND METHODS

### 2.1 Seawater sampling and microcosm construction

Four liters of seawater (0–10 m depth) was collected from a region adjacent to an offshore oil extraction platform located in Campo do Frade, Rio de Janeiro (latitude 21°53′150″S and longitude 39°52′880″N). Half of the total amount of water collected was artificially contaminated with 1% (v/v) crude oil extracted from the same platform (grade API 20.91). Thereafter, triplicate microcosms were constructed in Falcon tubes containing 50–60 ml of noncontaminated or contaminated water (i.e. a sufficient quantity of water to completely fill the tube). In total, 63 microcosms were constructed and samples were obtained up to 40 days of incubation for molecular analyses, as shown in Table 1. Incubation under high pressure and low temperature was performed in two hyperbaric chambers containing a cooling system (as explained below) at the COPPE/UFRJ Submarine Technology Laboratory. One of these chambers was purposely depressurized after 20 days of incubation and repressurized for another 20 days. The other group remained pressurized over the 40 days of incubation.

**Table 1:** Different microcosms used in this study

Microcosms (replicates x sampling time = 63) Treatment labels	Temperature 4°C	Temperature 22°C	Pressure 0.1 MPa	Pressure 22 MPa	Oil contamination	20 days/repressurization
I (3 × 1 = 3)						
II (3 × 3 = 9) W22		x	x			
III (3 × 3 = 9) WO22		x	x		x	
IV (3 × 3 = 9) W4	x		x			
V (3 × 3 = 9) WO4	x		x		x	
VI (3 × 3 = 9) WP4	x			x		
VII (3 × 3 = 9) WPO4	x			x	x	
VIII (3 × 1 = 3) WP4 40D	x			x		x
IX (3 × 1 = 3) WPO4 40D	x			x	x	x

All microcosms were constructed in triplicate. Microcosms I were those representing the water sample without any treatment, sampled at time zero (T0). The other microcosms were sampled at 10, 20, and 40 days of incubation. The microcosms depressurized after 20 days of incubation and repressurized for another 20 days were only sampled after 40 days incubation. The microcosms subjected to different treatments are labeled with the notation: W—water; P—with pressure of 22 MPa; O—contaminated with oil; 4 or 22—incubation temperature of 4°C or 22°C, respectively; D—pressurized and repressurized microcosms; 10, 20, 40—sampling time (days); 1, 2, or 3—replicate number. For examples, WPO4 40 1 indicates a microcosm that underwent 22 MPa pressure at 4°C, contaminated with oil, sampled after 40 days incubation and is the first replicate of three; W22 40 3 indicates a microcosm that underwent without pressure at 22°C, with no contamination with oil, sampled after 40 days incubation and is the third replicate of three.

### 2.2 Hyperbaric chambers

The different microcosms were incubated inside two hyperbaric chambers especially adapted for this study (Figure S1A). The adapted system comprised two hyperbaric chambers with the ability to control temperature and pressure throughout the experiments. The hyperbaric chambers were filled with water for pressurization and removal of air. Pressurization of the hyperbaric chambers was performed by a pneumatically operated hydraulic pump at a pressurizing rate of approximately 0.7 MPa/min, until a pressure of 22 MPa was reached. The pressure inside the chambers was measured by an analog gauge and a hydraulic accumulator was installed in the pressure line to ensure pressure stabilization of the chamber (Figure S1B). The two chambers containing the microcosms were then accommodated in a tank containing a mixture of water and glycol (to prevent the water from freezing). This tank had a serpentine‐based refrigeration system, with water temperature being controlled by a chiller (Figure S1A, B). The temperature inside the chambers was kept constant at 4°C throughout the experiments and was measured by two PT‐100 temperature sensors connected to a digital display. As a control of cross‐contamination between the water inside the hyperbaric chambers and that of the microcosms, a fluorescent dye (fluorescein, 0.01 mg/ml) was added to the water of the pressurizing system. The contamination could be detected visually by the color change (bright green) of the water inside the microcosms. When water in the microcosms was being sampled, the pressure inside the chambers was progressively decreased for sample removal.

### 2.3 DNA extraction and PCR amplification of bacterial 16S rRNA encoding gene (*rrs*)

The content of each microcosm was shaken to avoid missing anything attached to the sides of the Falcon tubes, filtered through a Millipore membrane (0.22 μm), and the total DNA was extracted using the FastDNA^®^ Spin Kit for Soil (BIO 101 Systems, Ohio, USA) and then stored at 4°C prior to PCR amplification. PCR amplification of the bacterial *rrs* gene was performed using the pair of primers F‐968 (5′ AAC GCG AAG AAC CTT AC 3′) and R‐1401 (5′ GCG TGT GTA CAA GAC CC 3′), as described in Nübel et al. (1996). A GC‐clamp was added to the forward primer (Muyzer, de Waal, & Uitterlinden, 1993). A 50 μl mixture containing 1 μl of DNA, 0.2 μmol/L of each primer, 0.8 μmol/L dNTP, 2.5 mmol/L MgCl_2_, 0.3 μl *Taq* DNA polymerase and 10 μl of 5X PCR buffer supplied by the manufacturer (Promega^®^, São Paulo, Brazil) was used for PCR amplification. The amplification conditions were as follows: an initial denaturation for 2 min at 94°C; 30 cycles consisting of 1 min at 94°C, 1 min at 48°C, 1 min at 72°C; and an extension for 5 min at 72°C. The products were analyzed by electrophoresis in 1.4% agarose gels, followed by ethidium bromide staining (0.2 μg/ml in 1X TAE buffer) (Sambrook, Fritsch, & Maniatis, 1989).

### 2.4 Denaturing gradient gel electrophoresis (DGGE) and statistical analyses

DGGE analysis was carried out using the Ingeny PhorU2 apparatus (Ingeny International BV, The Netherlands). PCR products were loaded onto 8% (w/v) polyacrylamide gels in 1X TAE buffer. Polyacrylamide gels contained a denaturing gradient of urea and formamide varying from 45% to 65%. The gels were run for 17 hr at 140 V and 65°C. After this period, they were soaked‐stained for 1 hr in SYBR Green I nucleic acid staining solution (1.000X concentrated; Molecular Probes, The Netherlands) and immediately photographed under UV light using the STORM apparatus (GE Healthcare Life Science, Chicago, USA). Dendrograms were constructed based on the presence and absence of bands with the unweighted pair group method with mathematical averages (UPGMA) and the Dice similarity coefficient using the GelCompare II software (Applied Maths, Sint‐Martens‐Latem, Belgium).

### 2.5 High‐throughput sequencing and data analysis

DNA obtained from the different microcosms was PCR‐amplified using the barcode primers 515F/806R (Caporaso et al., 2011), which target the V4 region of the 16S rRNA coding gene. Amplification, pooling, and purification were performed by Macrogen (South Korea). All nine treatments sampled at day 40 (microcosms in triplicate, except for one sample of WP4 40 and two samples of WPO4 40) were sequenced using a MiSeq Platform and the MiSeq Reagent kit v3 (Illumina, USA). All subsequent analyses were carried out with the software package QIIME (Quantitative Insights into Microbial Ecology toolkit) (Caporaso et al., 2010a). All sequences were quality‐filtered as described by Bokulich et al. (2013). The remaining sequences were clustered in operational taxonomic units (OTUs) at 100% sequence identity using CD‐HIT‐DUP (http://weizhong-lab.ucsd.edu). The same program was used for the subsequent removal of chimeras. Representative sequences from each group generated using CD‐HIT‐DUP were clustered into operational taxonomic units (OTUs) at 97% sequence identity using UCLUST (Edgar, 2010). Then, representative sequences from each OTU were aligned with the Greengenes Core Set (DeSantis et al., 2006) using PyNAST (Caporaso et al., 2010b). Taxonomy was assigned to sequences using the BLAST tool (Altschul et al., 1997). The OTU‐generated matrices were used to calculate species diversity using Chao1 estimators (Chao, 1987), the Shannon–Weaver diversity index (Shannon & Weaver, 1949) and Evenness‐E_H_ and Dominance (Simpson, 1949). Significant differences within diversity measures obtained from each treatment were determined using Tukey's pairwise comparisons *p* < .05) (Table 2). Finally, the OTU‐generated matrices were exported into PAST software (Hammer, Harper, & Ryan, 2001) for nonmetrical multidimensional scaling (NMDS) analyses. All sequences have been deposited at the EMBL Nucleotide Sequence Database under the accession numbers LT719161‐LT721897.

**Table 2:** Estimated OTUs, richness, and diversity indices based on the OTU‐generated matrices

Samples (triplicates)	OTUs	Richness (Chao 1)	Diversity (Shannon)	Equitability (Evenness‐E_H_)	Dominance
T0	118 ± 12.29^ab^ a	125.45 ± 16.4^a^	5.24 ± 0.69^ac^	0.76 ± 0.10^ac^	0.06 ± 0.03^ac^
W22 40	134 ± 21^a^	146.57 ± 23.67^a^	5.94 ± 0.37^a^	0.84 ± 0.03^a^	0.03 ± 0.01^a^
WO22 40	92.7 ± 6.8^ab^	109.05 ± 17.53^a^	3.34 ± 0.33^bc^	0.51 ± 0.06^bc^	0.21 ± 0.07^bc^
W4 40	118 ± 7.0^ab^	125.67 ± 5.25^a^	5.97 ± 0.28^a^	0.87 ± 0.03^a^	0.03 ± 0.01^a^
WO4 40	103.3 ± 9.24^ab^	115.7 ± 13.3^a^	4.15 ± 0.32^abc^	0.62 ± 0.04^abc^	0.13 ± 0.01^ab^
WP4 40D	111.7 ± 6.43^ab^	133.74 ± 10.75^a^	4.74 ± 0.24^ac^	0.70 ± 0.04^abc^	0.07 ± 0.01^ac^
WPO4 40D	120 ± 12.17^ab^	128.27 ± 12.06^a^	5.53 ± 0.67^a^	0.80 ± 0.09^ac^	0.04 ± 0.02^a^
WP4 40^b^	78	97.46	3.6	0.57	0.13
WPO4 40	113 ± 1.41	139.6 ± 1.89	4.62 ± 0.07	0.68 ± 0.01	0.07 ± 0.01

a Different letters indicate statistically significant differences based on Tukey's test (*p* < .05).

b The samples WP4 40 and WPO4 40 were not used for Tukey's test comparison as triplicate values were not available.

## 3 RESULTS

### 3.1 Structure of the bacterial communities

The structure of the bacterial communities from the different microcosms containing seawater contaminated or not with crude oil and submitted to different treatments (Table 1) were evaluated using 16S rRNA‐based PCR‐DGGE after 0, 10, 20 and/or 40 days of incubation. Three microcosms (WP4 40 2, WP4 40 3, and WPO4 40 3) were discarded from further analyses as they were contaminated with the fluorescent dye added to the interior of the hyperbaric chambers. Analysis of DGGE patterns yielded clear clustering of the within‐treatment replicates (*n *=* *3), confirming the reproducibility of the molecular approach used here.

**Figure 1:**
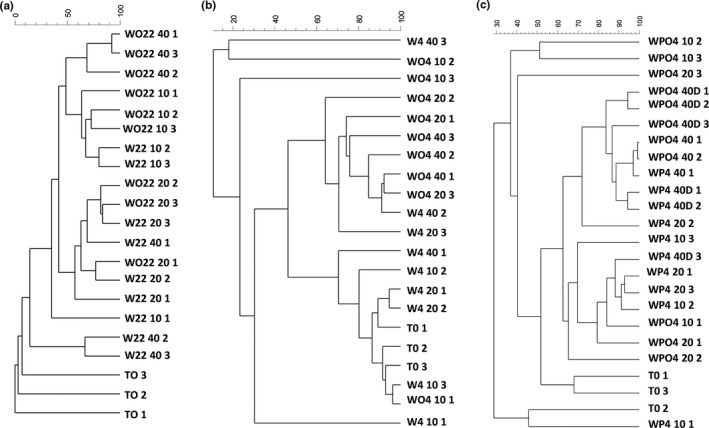
Dendrograms based on the DGGE profiles of the seawater microcosms using the Dice coefficient and the UPGMA clustering method. Microcosms were subjected to different treatments: (a) 22°C/0.1MPa; (b) 4°C/0.1MPa; (c) 4°C/22 MPa; and 4°C/22 MPaD. Samples are labeled as described in Table1

DGGE profiles from the 22°C/0.1 MPa treatment showed that the community structure varied between the beginning of the experiment (T0) and after 10, 20, and 40 days of incubation. The greatest number of bands was observed after 40 days of incubation and within the samples contaminated with oil (WO22 40, data not shown). UPGMA clustering analysis (Figure 1a) revealed that almost all profiles were separated according to whether they were contaminated with oil or not (similarity about 50%).

Under decreased temperature, that is, the 4°C/0.1 MPa‐treated microcosms, there were two groups of about 45% similarity (Figure 1b) constituting (A) microcosms without oil contamination, and (B) mainly oil‐contaminated samples, which followed the same pattern observed for microcosms at 22°C.

There were two main groups of 62% similarity for microcosms subjected to low temperature and high pressure (4°C/22 MPa) but, in this case, oil contamination did not seem to be the main factor influencing the community structure. For example, Group B was formed by samples incubated for 40 days with or without contamination. The longer exposure time to higher pressure seems to have contributed to altering the community structure (Figure 1c). The depressurization/repressurization step (4°C/22 MPaD) after 20 days of incubation did not result in a significant alteration of the structure of bacterial communities found in the seawater microcosms compared to the 4°C/22 MPa treatment (Figure 1c).

### 3.2 Diversity and composition of the bacterial communities

The diversity and composition of the bacterial communities in the different microcosms incubated for 40 days were determined using directly extracted community DNA. High‐throughput sequencing (Illumina MiSeq) of the V4 region of 16S rRNA resulted in up to 27,595 sequences (one WP4 40 replicate). The sequencing data were rarified to 4,500 reads per sample based on the lowest number of sequences obtained in one sample. OTU numbers and the diversity measures obtained from each treatment are shown in Table 2, and significant differences within the diversity values were determined using Tukey's pairwise comparisons (*p* < .05). The number of OTUs varied from 78 (WP4 40) to 155 (one W22 40 replicate) (Table 2). No significant difference was observed in bacterial richness (Chao1) among all treatments. Bacterial diversity (Shannon–Weaver index) and equitability were lower and dominance was higher in oil‐contaminated microcosms incubated under atmospheric pressure (22°C/0.1 MPa and 4°C/0.1 MPa) than in uncontaminated counterparts. In contrast, the microcosms contaminated with crude oil and submitted to high pressure and low temperature and also those depressurized and repressurized (4°C/22 MPa and 4°C/22MPaD, respectively) were more diverse (WPO4 40 and WPO4D 40; Table 2).

**Figure 2:**
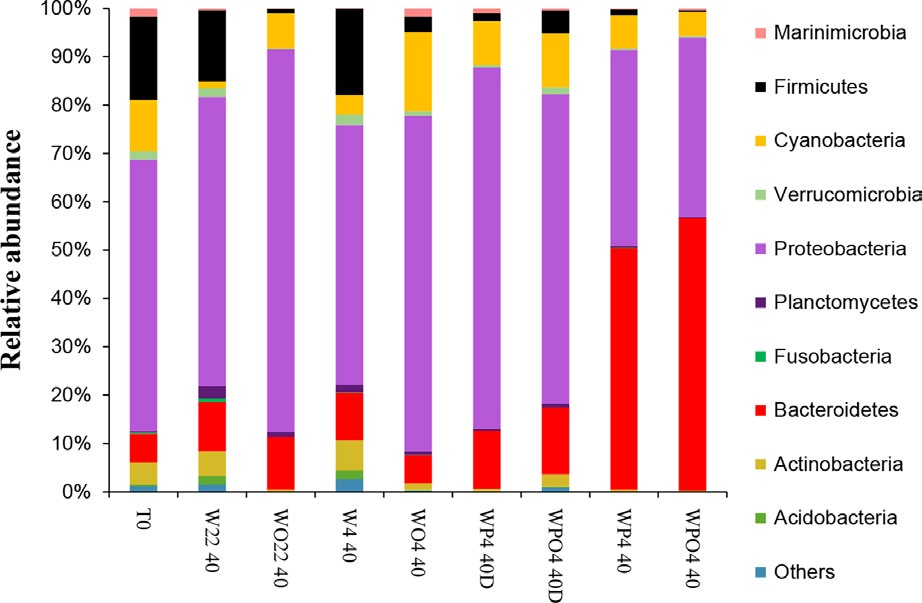
Relative abundances of the most abundant bacterial phyla found in the seawater samples, as determined by high‐throughput sequencing. Samples are labeled as described in Table1

Each replicate was considered individually for OTU analyses. As similar bacterial groups were found among replicates of the same treatment, we considered an average of the relative abundance of the different taxa found in the different microcosms incubated for 40 days. Only the taxonomic groups representing more than 5% of the relative abundance were considered for further analyses, with the remainder (less than 5% of the relative abundance) being denoted as “others”. The phylum Proteobacteria predominated in T0 and 22°C/0.1 MPa, 4°C/0.1 MPa, and 4°C/22 MPaD samples (Figure 2). For example, samples WO22 40 and WP4 40D presented relative abundances of 78.3 and 73.9% of Proteobacteria, respectively. Relative abundances of the phylum Firmicutes decreased with the presence of oil contamination either at 22°C or 4°C. Greater relative abundances of OTUs related to Bacteroidetes were observed for WP4 40 (49.4%) and WPO4 40 (55.8%). A slight difference in abundances of OTUs related to Bacteroidetes was observed in the depressurized/repressurized microcosms between those with and without oil (WPO4 40D and WP4 40D, respectively). OTUs related to Cyanobacteria, Actinobacteria, Verrumicrobia, and others were also found in different samples (Figure 2, Table S1).

**Figure 3:**
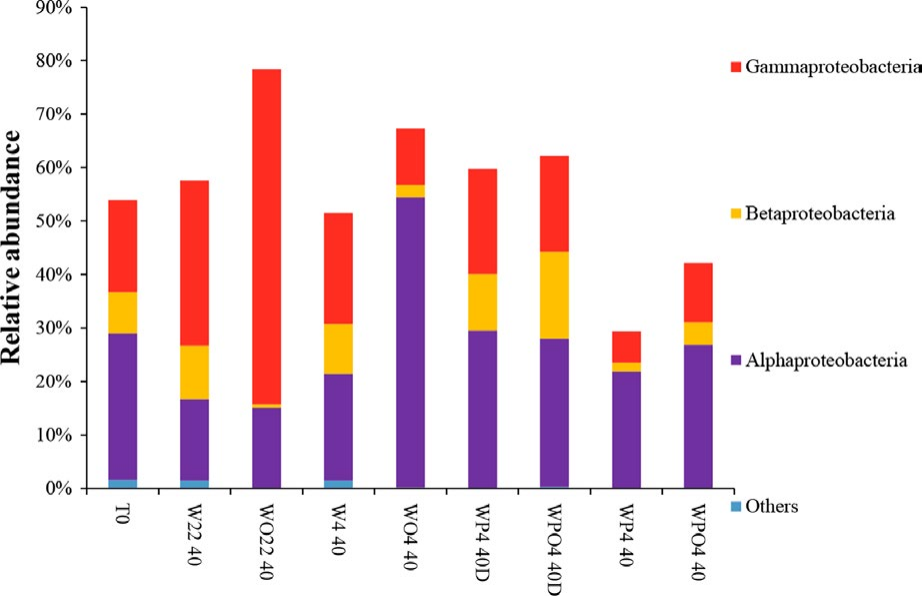
Relative abundances of the most abundant Proteobacteria classes found in the seawater samples, as determined by high‐throughput sequencing. Samples are labeled as described in Table1

Bacteria from different classes belonging to Proteobacteria were enriched in the microcosms contaminated or not with oil (Figure 3). The relative abundance of Gammaproteobacteria was 63% in WO22 40 and 31% in W22 40, respectively (a difference of 32%). Likewise, there was a difference of 34% in the relative abundances of Alphaproteobacteria between WO4 40 (54%) and W4 40 (20%) (Figure 3). Oppositely, the relative abundance of OTUs related to Betaproteobacteria and Gammaproteobacteria decreased with the addition of oil in microcosms incubated at 22°C and 4°C/0.1 MPa, respectively, when compared with their counterparts without oil contamination (Figure 3).

**Figure 4:**
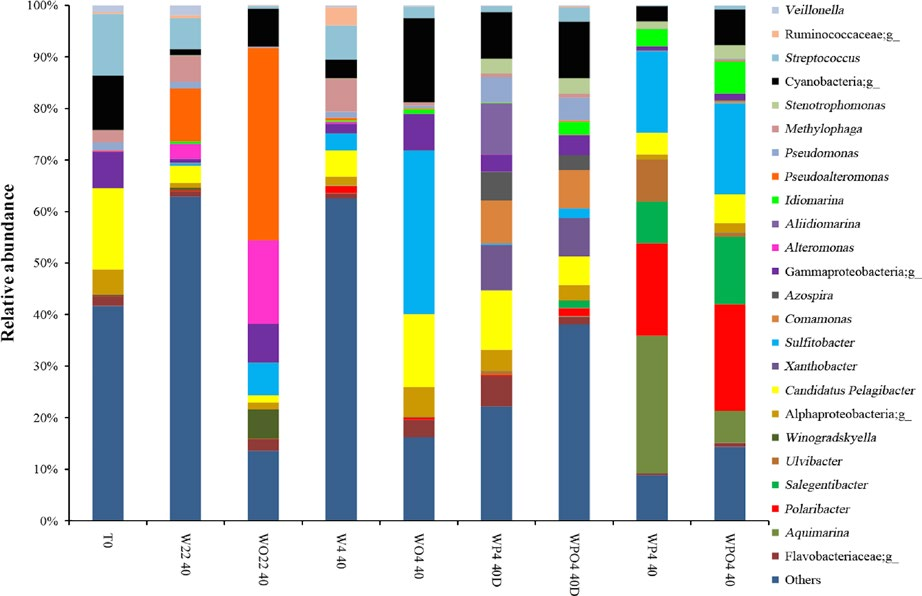
Relative abundances of the most abundant bacterial genera found in the seawater samples, as determined by high‐throughput sequencing. Samples are labeled as described in Table1

Specific groups of bacterial genera predominated according to the different treatments microcosms were subjected to. More than 40% of the bacterial communities found in T0 and noncontaminated samples (22°C and 4°C at 0.1 MPa) were represented by OTUs with relative abundances of less than 5% (Figure 4, Table S2). Oil‐contaminated microcosms incubated at 22°C/0.1 MPa exhibited different bacterial genera, such as *Pseudoalteromonas*,* Alteromonas*,* Sulfitobacter*,* Winogradskyella*, and others, all representing 0.1% or less of the relative abundance in T0 samples. The genera *Pseudoalteromonas* and *Alteromonas* showed increased relative abundances of 27% and 13%, respectively, when WO22 40 microcosms were compared to those of W22 40. Likewise, OTUs related to *Sulfitobacter* also showed an increase of almost 6% in relative abundance. Sequences related to this genus were not found in T0 samples (Figure 4, Table S2).

Considering the microcosms incubated at low temperature and at atmospheric pressure (4°C/0.1 MPa), oil contamination resulted mainly in the increased presence of *Sulfitobacter*. The relative abundance of sequences associated with this genus was 28% higher for WO4 40 compared to W4 40 microcosms. The second‐most frequent group of sequences in WO4 40 microcosms was assigned to Candidatus Pelagibacter. This group was found in T0 samples with a relative abundance of 15.3% (Figure 4, Table S2).

Little variation in relative abundance was observed for microcosms subjected to low temperature and high pressure (4°C/22 MPa), either for those contaminated (WPO4 40) or not (WP4 40) with crude oil. However, relative abundances of sequences related to different genera, such as *Aquimarina*,* Polaribacter*,* Salegentibacter*,* Sulfitobacter*,* Idiomarina*, substantially increased after 40 days of incubation (compared to T0, Figure 4). The samples depressurized after 20 days of incubation at 4°C and repressurized for another 20 days (WP4 40D and WPO4 40D) exhibited almost the same genera as for the WP4 40 and WPO4 40 microcosms. Moreover, there was little variation in the relative abundances of the sequences in oil‐contaminated and noncontaminated samples (Figure 4, Table S2).

### 3.3 NMDS of 16S rRNA‐based OTUs

**Figure 5:**
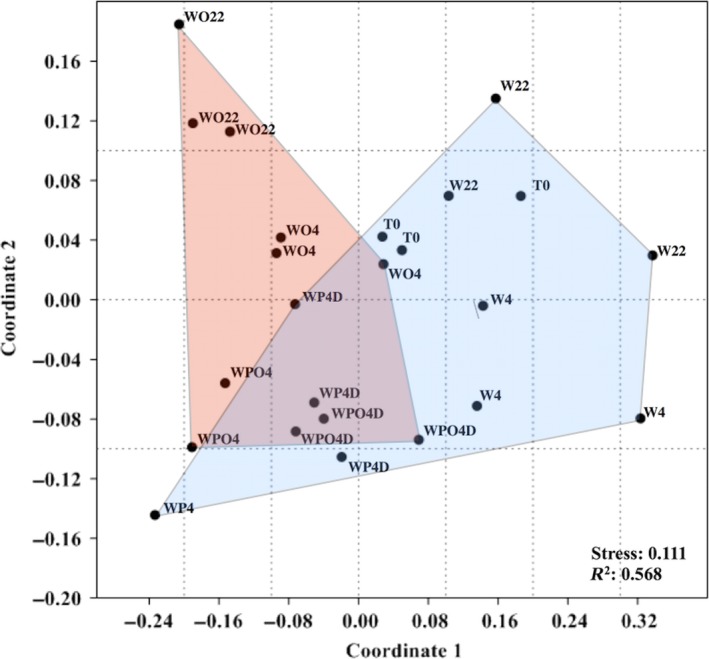
Nonmetric multidimensional scaling (NMDS) ordination diagram based on the OTU‐generated matrices obtained after sequencing using primers for 16S rRNA. Samples are labeled as described in Table 1 without the replicate number and the sampling time (after 40 days incubation)

NMDS analysis revealed a clear clustering of pressurized and nonpressurized seawater samples in a distinct region of the plot (Coordinate 1, Figure 5). Moreover, NMDS of 16S rRNA‐based OTUs revealed that oil‐contaminated samples could be distinguished from those of noncontaminated water samples by Coordinate 2 (Figure 5).

## 4 DISCUSSION

Understanding how seawater bacterial communities respond to hydrocarbon input and to decreased temperature and increased pressure while they migrate to the deep sea is essential for bioremediation purposes and also to find biomarkers of oil contamination. While different petroleum‐degrading marine bacteria have already been described (Head et al., 2006), studies on the effects of the low temperature and high pressure of deep sea environments have been hindered by the experimental logistics of testing these abiotic factors.

Parkes et al. (2009) developed a system (DeepIsoBUG) for the enrichment of bacteria from subsurface gas hydrate sediments from the Indian Continental Shelf. They observed that highest cell concentrations generally occurred close to 14 MPa in a variety of media, and that growth continued up to at least 80 MPa. More recently, Wannicke et al. (2015) described a high‐pressure incubation system in which the bacterial community composition of a decompressed sample from the Mediterranean deep sea (3,044 m) was determined. In our study, results were obtained using hyperbaric chambers adapted to maintain seawater samples (contaminated or not with oil) simultaneously at low temperature (4°C) and high pressure (22 MPa). The pressure we applied was based on the depth of the greatest reserves of oil and natural gas found in the presalt layer located below an ultradeep water column (greater than 2,000 meters) 800 km from the Brazilian coast (Filho, Pinto, & Almeida, 2011). The surface water was considered here because of logistic facilities and supported by the data presented in Frank et al. (2016) and Hubalek et al. (2016). These authors demonstrated the transport of water masses in the eastern North Atlantic and in a deep terrestrial subsurface aquifer of the Fennoscandian shield, respectively, and their influence on the dark ocean prokaryotic community composition.

Using molecular approaches, we have demonstrated the effect of oil contamination on bacterial communities in microcosms incubated for 40 days at either 4°C or 22°C under atmospheric pressure. Bacterial community structure changed more rapidly when the oil‐contaminated microcosms were incubated at 22°C. In contrast, oil‐contaminated and noncontaminated water samples incubated at 4°C and 22 MPa could not be separated by our PCR‐DGGE analyses. These results may be explained by the negative effect of low temperature and high pressure on microbial metabolism. Under extreme environmental conditions, metabolic potential decreases and the diversity of oil‐degrading bacteria is also diminished (Margesin & Schinner, 2001). For example, Scoma et al. (2016) have shown that the metabolism of hydrocarbonoclastic *Alcanivorax* spp. decreases as the environmental pressure increases. The same effect has been demonstrated in other oil‐degrading marine bacteria, such as *Rhodococcus qingshengii* and *Sphingobium yanoikuyae* (Schedler et al., 2014).

Nevertheless, we hypothesized that there are some groups of bacteria that tolerate high pressure and low temperature and grow in oil‐contaminated deep sea water. The question was whether the same bacterial genera are enriched or not in oil‐contaminated seawater under different temperatures and pressure. Our results showed that Bacteroidetes was the main bacterial phylum in 4°C/22 MPa‐treated microcosms, whereas Proteobacteria represented higher relative abundances in 22°C/0.1 MPa and 4°C/0.1 MPa treatments. Gammaproteobacteria was the predominant class in 22°C/0.1 MPa microcosms, as previously observed for other petroleum‐contaminated marine waters (Hazen et al., 2010; Jurelevicius et al., 2013; Liu & Liu, 2013; Mason et al., 2012). Alphaproteobacteria presented the class with the highest relative abundance in microcosms incubated under low temperature.

Although it is well established that after marine oil spills indigenous oil‐degrading bacteria (mostly belonging to Alphaproteobacteria or Gammaproteobacteria) increase their abundances (Yakimov, Timmis, & Golyshin, 2007), Gammaproteobacteria (but not Alphaproteobacteria as observed here) usually become enriched at low seawater temperatures (Dubinsky et al., 2013; Hazen et al., 2010). Moreover, different Gammaproteobacteria (such as *Pseudoalteromonas* and *Alteromonas*, whose relative abundances increased in the oil‐contaminated microcosms incubated at 22°C and at 0.1 MPa) and Alphaproteobacteria (such as *Sulfitobacter*, whose relative abundance increased in oil‐contaminated microcosms incubated at 4°C and at 0.1 MPa) have already been described as petroleum degraders (Gutierrez et al., 2013; Hazen et al., 2010; Jung et al., 2010; Meng et al., 2016), which may explain the enrichment of these bacteria in oil‐contaminated microcosms. Similarly, Liu, Bacosa, and Liu (2017) demonstrated that temperature is quite important for the selection of oil‐degrading bacteria, as they found that 4°C favored the development of *Cycloclasticus*,* Pseudoalteromonas*, *Sulfitobacter* (as observed here), and *Reinekea*, while 24°C incubations enhanced *Oleibacter*,* Thalassobius*,* Phaeobacter,* and *Roseobacter* from water samples collected near the Deepwater Horizon site.

The relative abundance of the OTUs for depressurized and then repressurized microcosms varied compared to those at the beginning of the experiment and also those pressurized continuously for 40 days. Abrupt changes in pressure can alter bacterial physiology and often lead to cellular lysis (Koyama, Kobayashi, Inoue, Miwa, & Aizawa, 2005). However, our results did not show the decrease in bacterial richness observed by Wannicke et al. (2015) where, after 3 days of incubation at 27 MPa, the natural bacterial deep sea community was dominated by bacteria of the genus *Exigucobacterium*. Moreover, Marietou and Bartlett (2014) observed that the pressure (80 MPa)‐induced community changes in Southern California coastal seawater included an increase in the relative abundance of Alphaproteobacteria, Gammaproteobacteria, Actinobacteria, and Flavobacteria largely at the expense of Epsilonproteobacteria. In our study, on the contrary, a decrease in the relative abundance of Gammaproteobacteria and also of Betaproteobacteria (to a lesser extent) was observed in the microcosms subjected to low temperature and high pressure (4°C/22 MPa).

Although depressurization generated an evident stress on the microbial community present in microcosms incubated at 4°C and at 22 MPa, the relative abundances of sequences related to different genera, such as *Aquimarina*,* Polaribacter*,* Salegentibacter*,* Sulfitobacter*,* Idiomarina* in both oil‐contaminated 4°C/22 MPa and 4°C/22 MPaD microcosms were increased. There are few studies on piezophilic or piezotolerant oil‐degrading bacteria. However, bacteria from the genera *Polaribacter*,* Salegentibacter* and/or *Idiomarina* have been described extensively in cold environments and as oil‐degraders (Wang, Zhang, Shan, & Shao, 2014).

In conclusion, this study provides a more complete understanding of the diversity of bacterial communities in seawater and their capacity to respond to an influx of crude oil in deep seawaters when subjected to different abiotic conditions. It is evident that hydrocarbon input was the main factor influencing the bacterial communities in the microcosms incubated at atmospheric pressure. Incubation of the microcosms in hyperbaric chambers especially adapted for this study showed that increased pressure was the factor that most influenced bacterial communities, and that specific bacterial taxa predominated depending on the temperature and pressure variations.

## CONFLICT OF INTEREST

The authors declare that they have no competing interests.
