# Supplementary material for: Response of marine bacteria to oil contamination and to high pressure and low temperature deep sea conditions
Source: Microbiologyopen. 2017 Oct 23;7(2):e00550. doi: 10.1002/mbo3.550 (PMC5912000; doi:10.1002/mbo3.550)
Supplement: Supplementary file 1 [file MBO3-7-na-s001.docx]

**Figure S1**. (A) Hyberbaric system during operation. (B) Schematic drawing of the hyberbaric system showing temperature and pressure components.


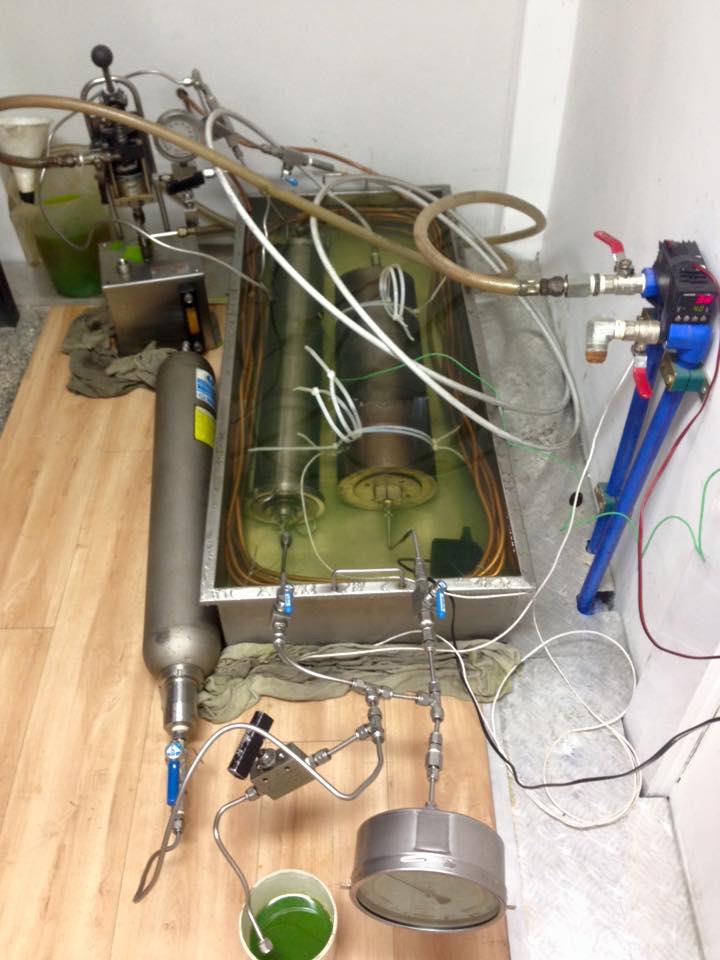

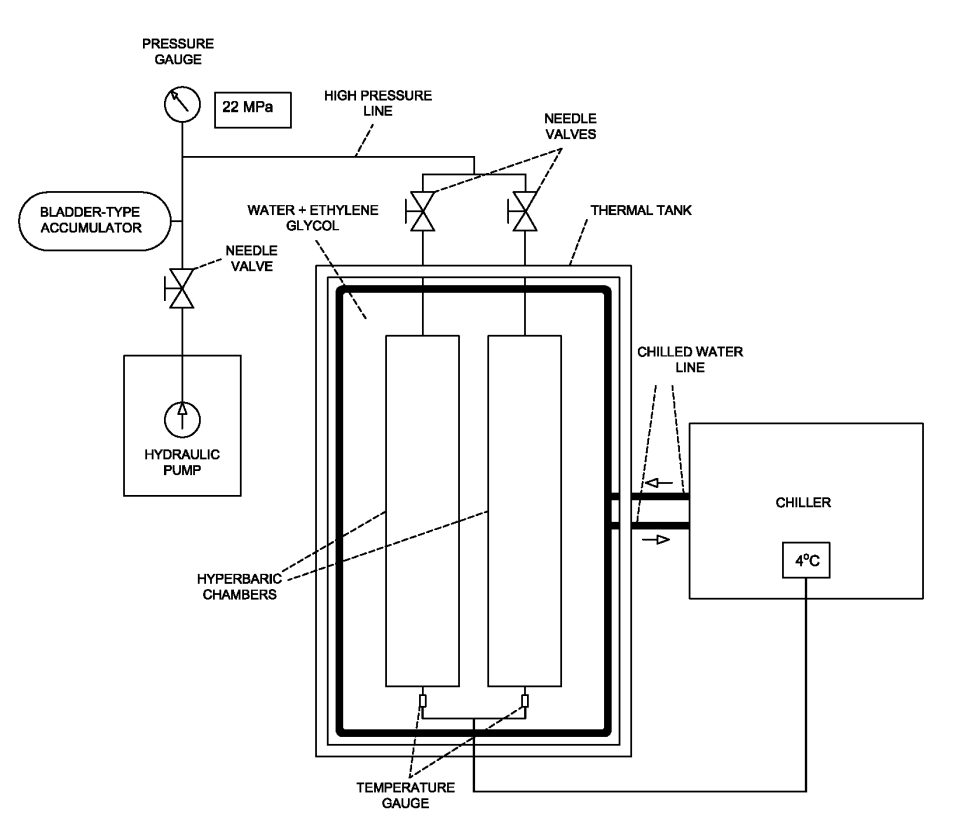


**B**

**A**

**Table S1**. Relative abundances (%) of the different phyla found in the different seawater microcosms (triplicates) submitted to different treatments

|  | T0 | W22 40 | WO22 40 | W4 40 | WO4 40 | WP4 40D | WPO4 40D | WP4 40 | WPO4 40 |
| --- | --- | --- | --- | --- | --- | --- | --- | --- | --- |
|  |  |  |  |  |  |  |  |  |  |
| *Others* | 5 ± 3 | 4 ± 1 | 0.0 | 2.5 ± 2 | 3 ± 1 | 0.1 | 3 ± 2 | 0.0 | 0.0 |
| Acidobacteria | 0.3 | 1.8 ± 1 | 0.0 | 1.8 ± 1 | 0.0 | 0.0 | 0.2 | 0.0 | 0.0 |
| Actinobacteria | 4.5 ± 1 | 4.9 ± 2 | 0.4 | 6.0 ± 2 | 1.4 ± 1 | 0.5 | 2.5 ± 1 | 0.4 | 0.3 |
| Bacteroidetes | 5.7 ± 2 | 9.7 ± 4 | 10.8 ± 9 | 9.4 ± 1 | 5.6 ± 1 | 11.9 ± 3 | 13.4 ± 4 | 49.4 | 55.8 ± 15 |
| Fusobacteria | 0.3 | 1 ± 1 | 0.0 | 0.1 | 0.1 | 0.0 | 0.0 | 0.0 | 0.0 |
| Planctomycetes | 0.3 | 2.4 ± 1 | 1.1 ± 1 | 1.5 | 0.7 | 0.4 | 0.7 | 0.3 | 0.2 |
| Proteobacteria | 53.9 ± 8 | 57.6 ± 10 | 78.3 ± 7 | 51.5 ± 4 | 67.2 ± 2 | 73.9±10 | 62.2 ± 11 | 40.1 | 36.8 ± 10 |
| Verrucomicrobia | 1.7 ± 1 | 1.7 ± 1 | 0.2 | 2.1 ± 1 | 0.9 | 0.4 | 1.4 ± 1 | 0.2 | 0.3 |
| Cyanobacteria | 10.2 ± 6 | 1.4 ± 1 | 7.3 ± 1 | 3.9 ± 5 | 15.9 ± 3 | 9.1 ± 5 | 10.8 ± 7 | 6.8 | 5 ± 3 |
| Firmicutes | 16.5 ± 9 | 14.2 ± 7 | 0.9 ± 1 | 17.1 ± 4 | 3 ± 2 | 1.6 ± 1 | 4.7 ± 2 | 1.2 | 0.4 |
| Marinimicrobia | 1.6 ± 1 | 0.3 ± 1 | 0.1 | 0.0 | 1.7 ± 1 | 1 ± 1 | 0.4 | 0.1 | 0.3 |

**Table S2.** Relative abundances (%) of the different genera found in the different seawater microcosms (triplicates) submitted to different treatments

|  |  | T0 | W22 40 | WO22 40 | W4 40 | WO4 40 | WP4 40D | WPO4 40D | WP4 40 | WPO4 40 |
| --- | --- | --- | --- | --- | --- | --- | --- | --- | --- | --- |
|  |  |  |  |  |  |  |  |  |  |  |
| Others | | 40.4 ± 16 | 61.8 ± 9 | 13.4 ± 2 | 61.1 ± 15 | 15.8 ± 5 | 22.0 ± 8 | 37.2 ± 16 | 8.8 | 14.1 ± 2 |
| Flavobacteriaceae | | 1.8 ± 1 | 1.1 ± 1 | 2.2 ± 1 | 1.0 ± 1 | 3.3 | 5.8 ± 4 | 1.5 ± 1 | 0.3 | 0.8 |
| *Aquimarina* |  | 0.0 | 0.0 | 0.0 | 0.0 | 0.0 | 0.0 | 0.1 | 26.6 | 6.2 ± 2 |
| *Polaribacter* |  | 0.1 | 0.2 | 0.2 | 1.0 ± 2 | 0.4 | 0.2 | 1.6 ± 1 | 17.9 | 20.4 ± 3 |
| *Salegentibacter* | | 0.0 | 0.0 | 0.0 | 0.0 | 0.0 | 0.0 | 1.4 ± 1 | 8.0 | 12.9 |
| *Ulvibacter* |  | 0.0 | 0.0 | 0.0 | 0.2 | 0.0 | 1.0 ± 1 | 0.0 | 8.1 | 0.7 ± 1 |
| *Winogradskyella* | | 0.2 | 0.0 | 6.0 ± 1 | 0.0 | 0.1 | 0.0 | 0.1 | 0.0 | 0.0 |
| Alphaproteobacteria | | 4.7 ± 2 | 1.0 ± 1 | 1.3 | 2.0 ± 2 | 5.6 ± 2 | 4.1 ± 3 | 2.9 ± 1 | 1.1 | 1.8 |
| *Candidatus Pelagibacter* | | 15.3 ± 7 | 3.3 ± 3 | 1.5 | 5.0 ± 3 | 13.7 ± 3 | 11.4 ± 7 | 5.4 ± 1 | 4.1 | 5.6 ± 1 |
| *Xanthobacter* | | 0.0 | 0.0 | 0.0 | 0.0 | 0.0 | 8.8 ± 6 | 7.3 ± 8 | 0.0 | 0.0 |
| *Sulfitobacter* |  | 0.0 | 0.5 | 6.3 ± 3 | 3.0 ± 4 | 30.9 ± 1 | 0.2 | 1.8 ± 1 | 15.8 | 17.4 ± 2 |
| *Comamonas* |  | 0.0 | 0.0 | 0.0 | 0.0 | 0.0 | 8.3 ± 6 | 7.3 ± 4 | 0.1 | 0.4 |
| *Azospira* |  | 0.0 | 0.0 | 0.0 | 0.0 | 0.0 | 5.5 ± 4 | 2.8 ± 3 | 0.0 | 0.2 |
| Gammaproteobacteria | | 6.8 ± 2 | 1.0 ± 1 | 7.4 ± 11 | 1.7 ± 1 | 6.9 ± 1 | 3.3 ± 2 | 3.7 ± 2 | 0.8 | 1.1 |
| *Alteromonas* |  | 0.1 | 3.0 ± 3 | 16.0 ± 9 | 0.0 | 0.0 | 0.0 | 0.1 | 0.0 | 0.0 |
| *Aliidiomarina* | | 0.0 | 0.0 | 0.0 | 0.0 | 0.0 | 9.9 ± 5 | 0.0 | 0.0 | 0.0 |
| *Idiomarina* |  | 0.0 | 0.0 | 0.0 | 0.0 | 0.8 | 0.1 | 2.5 ± 1 | 3.3 | 6.2 |
| *Pseudoalteromonas* | | 0.1 | 10.1 ± 9 | 36.9 ± 11 | 0.0 | 0.2 | 0.0 | 0.3 | 0.1 | 0.1 |
| *Pseudomonas* | | 2.0 ± 2 | 1.3 ± 1 | 0.1 | 1.0 ± 1 | 0.0 | 4.9 ± 3 | 4.3 ± 1 | 0.1 | 0.4 |
| *Methylophaga* | | 2.0 ± 2 | 4.9 ± 2 | 0.2 | 6.3 ± 3 | 1.0 | 1.0 | 0.7 | 0.0 | 0.0 |
| *Stenotrophomonas* | | 0.0 | 0.2 | 0.0 | 0.0 | 0.0 | 2.9 ± 2 | 3.0 ± 3 | 1.3 | 2.6 |
| Cyanobacteria | | 10.2 ± 6 | 1.2 ± 1 | 7.3 ± 1 | 4.0 ± 4 | 15.9 ± 3 | 9.0 ± 6 | 10.8 ± 7 | 3.0 | 6.8 |
| *Streptococcus* | | 11.6 ± 10 | 5.9 ± 1 | 1.0 | 6.4 ± 2 | 2.1 ± 1 | 1.2 ± 1 | 2.6 ± 1 | 0.1 | 1.0 |
| Ruminococcaceae | | 0.0 | 0.0 | 0.1 | 3.4 ± 3 | 0.1 | 0.0 | 0.3 | 0.0 | 0.0 |
| *Veillonella* |  | 1.3 ± 1 | 2.0 ± 3 | 0.0 | 0.0 | 0.1 | 0.0 | 0.1 | 0.0 | 0.0 |
